# Pedal edema with olanzepine

**DOI:** 10.4103/0253-7613.48883

**Published:** 2009-02

**Authors:** Veena Nayak, Bharti Chogtu, Virupaksha Devaramane, P. V. Bhandary

**Affiliations:** Department of Pharmacology, Kasturba Medical College, Madhav Nagar, Manipal, Karnataka, India; 1 Department of Psychiatry, Dr. A. V. Baliga Memorial Hospital, Udupi, Karnataka, India

**Keywords:** Olanzapine, pedal edema

## Abstract

Olanzapine, an atypical antipsychotic is considered superior to its conventional congeners. Here we report two cases of pedal edema secondary to olanzapine. In both cases the systemic causes of pedal edema were ruled out. On reducing the dose of olanzapine, pedal edema regressed and completely resolved after stopping the drug. So we attribute the edema to olanzapine therapy. As the definitive cause and further consequences of pedal edema are not known, hence stringent monitoring of adverse effects of this drug is required.

## Introduction

Olanzapine, an atypical antipsychotic is widely used in the treatment of psychiatric illnesses like schizophrenia, bipolar affective disorder etc. It is superior to haloperidol in controlling the negative symptoms of schizophrenia. The drug acts by antagonizing the serotonergic (5HT2A), dopaminergic (D2, D1, D4), muscarinic (M1) and histaminergic (H1) receptors.[1] Olanzapine is started with a dose of 5 to 10 mg per day. The most common side effects of this drug are weight gain and somnolence.[2] Other side effects include dry mouth, dizziness, constipation, dyspepsia, increased appetite, akathesia and tremors. In premarketing trials, peripheral edema was reported as an infrequent side effect, which affected 3% of the 532 olanzapine treated patients, as compared to 1% of the 294 subjects on placebo.[3] We present two cases of pedal edema due to olanzapine, which we came across in a private hospital.

## Case Reports

### Case 1

A 37-year-old male patient presented with withdrawal symptoms due to alcohol. There was history of patient muttering to self, aggressive behavior, frequent job changes, marital disharmony, referential ideas and irritability. His withdrawal symptoms were treated with tab. lorazepam 8 mg on day 1, which was gradually tapered and stopped over seven days. After the withdrawal symptoms subsided, he was prescribed tab olanzapine 7.5 mg at bed time, to control his psychotic symptoms. The patient was in the hospital for eleven days and then discharged with the same treatment. He was advised to report for follow up once in 15 days. During the follow up, he complained of occasional fearfulness, ideas of reference and auditory hallucinations. After one and half months of starting the treatment, the patient complained of bilateral pedal edema. On examination, there were no positive findings which could be attributed to pedal edema. There was no history suggestive of thyroid dysfunction. Renal function tests, liver function tests and cardiac parameters were within normal limits.

Review of literature revealed a few case reports of pedal edema due to olanzapine. On this basis, the dose of olanzapine was tapered and stopped in ten days. The edema subsided and disappeared over 20 days. The patient was then treated with risperidone 2 mg per day, for the psychotic symptoms. On subsequent follow up, the edema did not recur.

### Case 2

A 30-year-old male patient reported to the out patient department with complaints of suspiciousness, fearfulness, withdrawn behavior, social avoidance, poor personal hygiene and hallucinations, since two years. He was diagnosed to have undifferentiated schizophrenia. All his baseline investigations were normal. Complete blood count, random blood sugar and renal function tests were normal. The patient was started on olanzapine 5 mg per day, which was gradually increased to 15 mg per day. He was in the hospital for twenty days and was then discharged with the same treatment. The patient came for follow up, two weeks after discharge; his condition had improved. He had started attending his job regularly. After two months, he complained of swelling of feet, which was more on the left foot than on the right. Olanzapine was tapered and stopped, as no other cause could be attributed. The edema disappeared over 15 days. He was then prescribed risperidone 2 mg per day and on subsequent follow up, it was found that the edema did not recur.

## Discussion

In both the cases discussed above, the edema can be attributed to olanzepine therapy, since the edema regressed when olanzapine was tapered and stopped. This also supports the earlier reports that it could be a dose related phenomenon.^4^ As per the causality assessment, olanzapine as a causative agent for edema can be considered probable (Naranjo's algorithm, score = 7).[5] In a case report of edema due to olanzapine, furosemide was added to treat the edema.[6] However, since this is a self limiting side effect, it does not require any intervention. Literature reports bilateral eyelid edema with olanzapine.[7]

To explain the edema due to olanzapine, various hypotheses have been put forth. Firstly, the edema can be attributed to vasodilation and decrease in vascular resistance, which is secondary to blockade of α_1_ receptors by olanzapine. What can also lead to vasodilation is 5HT_2_ blockade, through increasing cyclic adenosine monophosphate (cAMP).[4] Secondly, dopaminergic blockade due to olanzapine can alter the renal regulation of fluid and electrolytes, which play a role in producing edema.[8] Since other antipsychotics also share the same mechanisms, it is necessary to rigorously monitor the adverse events of this group as a whole.

As per previous reports and our two case reports, we would like to conclude that edema due to olanzapine is not an infrequent side effect. Since this edema does not interfere with the underlying disease process and is self limiting, it is often overlooked by clinicians.
